# Dispersal and population structure at different spatial scales in the subterranean rodent *Ctenomys australis*

**DOI:** 10.1186/1471-2156-11-9

**Published:** 2010-01-28

**Authors:** Matías S Mora, Fernando J Mapelli, Oscar E Gaggiotti, Marcelo J Kittlein, Enrique P Lessa

**Affiliations:** 1 Laboratorio de Ecofisiología, Departamento de Biología, Facultad de Ciencias Exactas y Naturales, Universidad Nacional de Mar del Plata. Casilla de Correo 1245, Funes 3250, 3erpiso. 7600 Mar del Plata, Argentina; 2 Laboratoire d'Ecologie Alpine (LECA), UMR CNRS 5553, Université Joseph Fourier, Grenoble, France; 3 Laboratorio de Evolución, Facultad de Ciencias, Universidad de la República, Montevideo 11400, Uruguay

## Abstract

****Background**:**

The population genetic structure of subterranean rodent species is strongly affected by demographic (e.g. rates of dispersal and social structure) and stochastic factors (e.g. random genetic drift among subpopulations and habitat fragmentation). In particular, gene flow estimates at different spatial scales are essential to understand genetic differentiation among populations of a species living in a highly fragmented landscape. *Ctenomys australis *(the sand dune tuco-tuco) is a territorial subterranean rodent that inhabits a relatively secure, permanently sealed burrow system, occurring in sand dune habitats on the coastal landscape in the south-east of Buenos Aires province, Argentina. Currently, this habitat is threatened by urban development and forestry and, therefore, the survival of this endemic species is at risk. Here, we assess population genetic structure and patterns of dispersal among individuals of this species at different spatial scales using 8 polymorphic microsatellite loci. Furthermore, we evaluate the relative importance of sex and habitat configuration in modulating the dispersal patterns at these geographical scales.

**Results:**

Our results show that dispersal in *C. australis *is not restricted at regional spatial scales (~ 4 km). Assignment tests revealed significant population substructure within the study area, providing support for the presence of two subpopulations from three original sampling sites. Finally, male-biased dispersal was found in the Western side of our study area, but in the Eastern side no apparent philopatric pattern was found, suggesting that in a more continuous habitat males might move longer distances than females.

**Conclusions:**

Overall, the assignment-based approaches were able to detect population substructure at fine geographical scales. Additionally, the maintenance of a significant genetic structure at regional (~ 4 km) and small (less than 1 km) spatial scales despite apparently moderate to high levels of gene flow between local sampling sites could not be explained simply by the linear distance among them. On the whole, our results support the hypothesis that males disperse more frequently than females; however they do not provide support for strict philopatry within females.

## Background

The characterization of population genetic structure and dispersal patterns offers fundamental information on the ecology and evolution of species and provides vital knowledge for the conservation of threatened species [1]. In such a context, dispersal is critical for long-term population viability because it can lead to the demographic and genetic rescue of small populations [2]. Dispersal is a complex phenomenon that has population level consequences but it originates as a behavioural trait at the level of individuals [3]. It is determined by sex and age of individuals as well as behavioural and social factors and their interaction with the geographical features of the landscape. Therefore, a thorough understanding of the demographic and genetic consequences of dispersal requires more than the simple estimation of migration rates. In particular, it is necessary to study how landscape features could influence movements at different spatial scales, and to determine how dispersal behaviour is influenced by both sex and age of individuals [4].

Fully characterising dispersal patterns is particularly difficult in the case of solitary species with territorial behaviour living in complex landscapes in which spatial structure can be described as a more or less continuous habitat, irregularly interrupted by discontinuities [4,5]. Many species of subterranean rodents fit in this description and represent a real challenge to the study of migration patterns. In general, these species occupy fragmented habitats and present limited dispersal abilities in relation to the spatial scale of the habitat discontinuities [3]. They occupy small population units with low genetic variation and high inter-population divergence [6]. This is the case of the subterranean rodents of the genus *Ctenomys *(tuco-tucos), which present restricted mobility and are usually distributed in patches with low local effective population numbers [6,7].

Like most species of tuco-tucos, the sand-dune tuco-tuco (*Ctenomys australis*) is solitary, highly territorial, and most likely polygynous [8]. Individuals build large burrow systems in coastal sand dunes along the Atlantic coast of the Buenos Aires province, Argentina [8-11]. *C. australis *is one of the largest species within the genus (250-500 g) and the extreme energetic costs associated with digging restrict the availability of suitable habitat for this species. The capacity of this species to remove soil decreases dramatically in harder substrates [7]. Hence, in comparison to other *Ctenomys *species [7,12], *C. australis *is currently restricted to a much reduced range of approximately 100 km^2 ^[11] and is strongly associated to the narrow coastal dunes [10].

The evolutionary history of *C. australis *has been markedly linked to the recent Quaternary sand dune origin and the geomorphologic evolution of the coastal plain in the Buenos Aires province [10]. Along the coast, the habitat of *C. australis *is practically linear and mostly continuous over the totality of its highly restricted distributional range (less than 270 km), only interrupted by small towns and streams, and a large river, the Quequén Salado River [10,11]. This nearly one-dimensional pattern of distribution along the coast imposes important restrictions on gene flow, and more generally, on the dynamic of differentiation within this species [10].

However, at a smaller spatial scale (less than 5 km), the habitat is recurrently interrupted by low inter-dune grasslands with harder soils [9,10]. Thus, at this scale, the habitat structure is rather heterogeneous with numerous effective potential barriers to gene flow that result in several isolated clusters of individuals along the coast [11].

In a previous study, Zenuto & Busch [8] implemented a removal design with posterior recapture of colonizing individuals to examine the age-structure of migrant individuals of this species. These authors suggest that dispersal between local populations seems to occur above ground; they found that colonizing groups were mainly composed of immature individuals and their sex ratio was similar to that observed in the local source populations. However, there is little information about the geographical scale at which dispersal takes place among different subpopulations in *C. australis*.

In particular, natal dispersal, which occurs in most subterranean rodents, tends to be sex biased and has important effects on social structure, population demography and genetic composition [6,12], particularly for small and isolated populations [2,4]. Therefore, estimates of relative dispersal rates between sexes in *C. australis *represent important information for guiding conservation plans [5].

The sand-dune habitat of *C. australis *is currently being lost to urban development, forestry and the progressive advance of grasslands, so survival of this endemic species is also linked to the preservation of the whole sand-dune ecosystem [10]. In order to design and implement measures for the preservation of this rodent and its habitat it is necessary to know more about the way in which habitat loss affects its population dynamics and genetics. For this reason, the study of the limits and extension of populations is essential for an appropriate determination of the conservation scale.

In general, little is known about dispersal in subterranean rodents because of the difficulties associated with direct quantification in species that live underground [3,7]. However, modern population genetics approaches can overcome the difficulties associated with mark-release-recapture methods [13] and can provide new insight into the dispersal abilities of subterranean rodents. In particular, many Bayesian clustering algorithms for inferring whether there is substructure by clustering individuals have been recently developed (e.g. see [14,15]). These methods use the genetic information contained in multilocus genotypes to ascertain population membership of the individuals sampled without assuming predefined populations (e.g. [16,17]). Individuals that are not assigned to the population in which they were sampled can be considered as migrants allowing direct estimation of migration rate [14]. Here, we use multilocus genotype data based on microsatellite markers and several recent Bayesian approaches to characterize the population structure and patterns of movements at different spatial scales in *C. australis*. Our objective is to determine to what extent the sex of individuals and landscape features influence dispersal patterns.

## Results

### **Habitat fragmentation**

**Figure 1:**
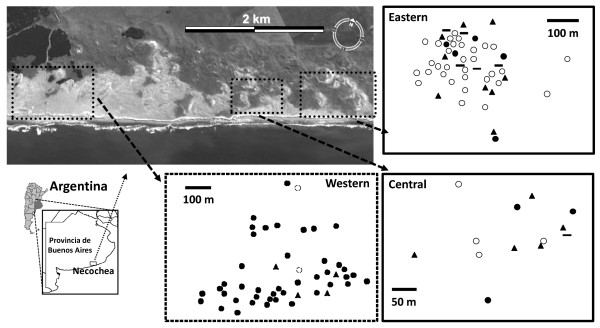
**Geographic distribution of *Ctenomys australis *sampling sites along the study area on the coast**. Squares over the map show the surface of each of the three sample sites (Western, Central and Eastern) and their individual points of capture within them (black circles: individuals pertaining to the Western genetic cluster; white circles: individuals pertaining to the Eastern genetic cluster; lines: extra-cluster immigrants; triangles: ambiguous individuals).

The mean fragmentation index was greater in the Eastern and Central sampling sites (0.027 and 0.028, respectively) than in the Western site (0.021). Both the Eastern and Central sites showed highly significant differences in the degree of habitat fragmentation in comparison to the Western site (P < 0.001; see Figure 1). On the other hand, differences between Eastern and Central sampling sites were not significant (P = 0.364).

### Genetic variability

Number of alleles per locus, number of alleles per sampling sites, observed heterozygosity and expected heterozygosity by locus for each geographical sample (Eastern, Central and Western) are presented in Table 1. Microsatellite allele frequencies are shown in Table 2. Only one locus (Hai9) was presumed to have null alleles and was therefore excluded from the analyses. The remaining eight loci were polymorphic with the total number of alleles per locus ranging between 3 and 6 (Table 1). Site C was fixed for one locus (Soc1) while sites E and W were polymorphic for all the loci studied. The total number of alleles was similar for all sampling sites, varying from 30 to 35 (Table 1). Two loci showed significant deviations from Hardy-Weinberg equilibrium within the Eastern site, and only one locus within the Western site (P < 0.05; Table 1). All these deviations were caused by an excess of heterozygotes. There were between 3 and 5 pair-wise disequilibrium tests per population that were significant, but none of them were significant after applying the Bonferroni correction for multiple tests. These results suggest that loci are independent.

**Table 1:** Microsatellite genetic variation in *C. australis*.

		Eastern			Central			Western		
***Locus***	**At**	**N*i***	**H*o***	**H*e***	**N*i***	**H*o***	**H*e***	**N*i***	**H*o***	**H*e***

**Soc1**	3	3	0.1	0.09	1	--	--	2	0.21	0.19

**Soc2**	6	5	0.69	0.74	6	0.85	0.76	6	0.69	0.76

**Soc5**	6	6	0.69	0.62	4	0.46	0.48	5	0.6	0.71

**Soc6**	5	4	0.37	0.35	3	0.54	0.43	3	0.4	0.33

**Soc8**	4	4	0.86*	0.66	3	0.85	0.65	3	0.73	0.59

**Hai3**	6	6	0.84*	0.61	3	0.62	0.45	5	0.63	0.53

**Hai4**	5	4	0.75	0.65	5	0.62	0.58	4	0.83*	0.65

**Hai11**	3	3	0.57	0.55	2	0.23	0.21	2	0.38	0.41

**Mean**			0.61	0.53		0.6	0.51		0.56	0.52

**A**	4.75	4.38			3.38			3.75		

**%P**		100			87.5			100		

**N_♂_**		28			7			17		

**N_♀_**		23			6			31		

**N_SSA_**		4			2			7		

**N_SA-A_**		47			11			41		

**N_T_**		51			13			48		

Number of alleles per locus (At), number of alleles per population (N*i*), observed heterozygosity (H*o*) and expected heterozygosity (H*e*) by locus for each geographical sample (Eastern, Central and Western). --, Monomorphic; Mean, average over eight loci; A, average number of alleles (allelic richness); %P, percentage of polymorphic loci. N_♂_, male sample size; N_♀_, female sample size; N_SSA_, smaller subadults sample size (between 160 to 210 g); N_SA-A_, larger subadults and adults sample size (bigger than 210 g); N_T_, total sample size. All individuals belong to a post-dispersal age [8,11]. Significant deviations between observed and expected levels of heterozygosity in each geographical sample and locus by locus are shown. * 0.001 < P < 0.05; and ** P < 0.001.

**Table 2:** Microsatellite allele frequencies across loci and sampling sites.

Locus/alleles	Sampling sites		
	Eastern	Central	Western
**Soc1**			

1	0.029	0.000	0.104

2	0.951	1.000	0.896

3	0.020	0.000	0.000

**Soc2**			

1	0.078	0.038	0.125

2	0.255	0.192	0.219

3	0.059	0.115	0.125

4	0.304	0.308	0.385

5	0.304	0.308	0.115

6	0.000	0.038	0.031

**Soc5**			

1	0.029	0.000	0.000

2	0.157	0.192	0.177

3	0.088	0.038	0.115

4	0.069	0.077	0.125

5	0.578	0.692	0.469

6	0.078	0.000	0.115

**Soc6**			

1	0.147	0.154	0.000

2	0.049	0.115	0.177

3	0.000	0.000	0.021

4	0.010	0.000	0.000

5	0.794	0.731	0.802

**Soc8**			

1	0.235	0.308	0.104

2	0.412	0.423	0.448

3	0.343	0.269	0.448

4	0.010	0.000	0.000

**Hai3**			

1	0.069	0.000	0.073

2	0.029	0.038	0.000

3	0.539	0.692	0.646

4	0.020	0.000	0.021

5	0.294	0.269	0.219

6	0.049	0.000	0.042

**Hai4**			

1	0.000	0.115	0.198

2	0.010	0.038	0.281

3	0.353	0.077	0.042

4	0.431	0.615	0.479

5	0.206	0.154	0.000

**Hai11**			

1	0.069	0.000	0.000

2	0.392	0.885	0.708

3	0.539	0.115	0.292

### Population structure and gene flow

The largest pair-wise *F*_ST _was observed between the two most distant sampling sites (E and W) but the central one was more similar to the more distant Western site than to the Eastern (Table 3). Thus, at this scale there is no simple relation between geographic distance and pair wise *F*_ST_s. The local population *F*_ST _was largest for the Western site and lowest for the Eastern site being intermediate for the central one (Table 3). These results suggest stronger genetic drift and/or lower immigration rate for the Western sampling site than for the two other sampling sites.

**Table 3:** Genetic differentiation in *Ctenomys australis*.

Population pairwise comparisons	Eastern sample	Central sample	Western sample	Local *F*_ST_
**Eastern sample**	---	0.053* (9.5)	0.059* (8.53)	0.0604

**Central sample**	0.051*	---	0.029* (17.28)	0.0814

**Western sample**	0.057*	0.029*	---	0.100

Weir & Cockerham ([45]; below the diagonal) and Slatkin ([46]; above the diagonal) *F*_ST _values for pairwise comparisons. Effective numbers of migrants per generation (*N*m, according to Slatkin method) are also reported in parentheses above the diagonal. * P < 0.05. Local *F*_ST _express local differentiation in relation to the whole population as described by Foll & Gaggiotti [47].

The global Mantel test based on between-individual geographic and genetic distances did not uncover a relationship between genetic and geographic distances (r = -0.007, P > 0.615). These results are in accordance with the pair wise *F*_ST_'s and suggest a lack of an overall isolation by distance pattern at the regional spatial scale covered by our study (4 km).

**Figure 2:**
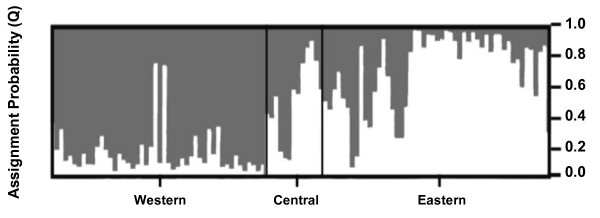
**Posterior assignment probabilities**. Assignment probabilities (Q) to Western (dark grey) and Eastern (white) genetic clusters (K = 2), derived from the STRUCTURE analysis. Each individual is represented by a vertical bar.

**Figure 3:**
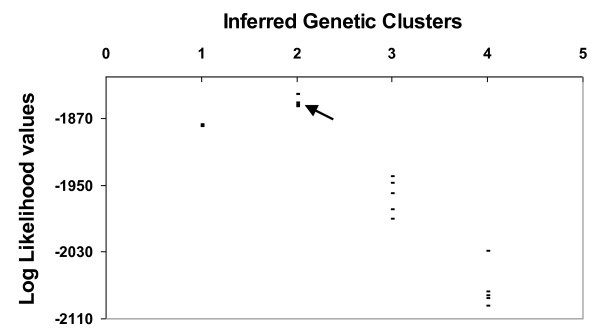
**Inference of genetic clusters (K) from *Ctenomys australis *sampling sites of Necochea (Argentina)**. The log-likelihood values (points) based on the STRUCTURE algorithm, for each 6 independent runs to the whole data set (n = 112) are also shown. The black arrow shows the best clustering solution.

The Bayesian analysis using STRUCTURE clearly identified the presence of substructure among the sampling sites; the model that best fit the data was K = 2. All runs at K = 2 produced identical clustering solutions with similar values of cluster membership (Q) for all individuals (Figure 2 and 3). One of these clusters consisted of 84.2% of individuals belonging to sampling site E, 10.5% belonging to site C and 5.3% belonging to site W. The second cluster comprised 84% of individuals from sampling site W, 10% of individuals from site E and 6% of individuals from site C. In order to avoid ambiguities, we will use the term site for referring to the sampling locations and the term genetic cluster for referring to the clusters identified by STRUCTURE. These results show a good agreement between the genetic clusters identified by STRUCTURE and the geographic location of the samples, with an Eastern subpopulations corresponding to cluster 1 and a Western subpopulation corresponding to cluster 2. The individuals belonging to the central sampling site, with a high percentage of admixed genotypes, were assigned in similar proportions to each one of the others two clusters (Figure 2). This genetic structuring identified by STRUCTURE at this regional scale was also confirmed by analyses carried out with TESS.

There were a total of 24 individuals that could not be assigned to either genetic cluster using the threshold value of Q > 0.7; 14 of these were from site E, 6 from site C and 4 from site W. Six of them were identified as individuals that did not belong to any of the two clusters using the GeneClass exclusion test. The remaining 18 individuals could neither be assigned to a genetic cluster nor excluded from both of them; therefore, they were not taken into consideration for the inference of migration patterns.

The six individuals that were excluded from both clusters were considered as immigrants that came from unsampled populations. One of them was sampled in site W and the other five in site E. The average genetic distance among these six individuals (GD = 50.13) was not significantly different from that obtained for the western genetic cluster (GD = 51.54; P = 0.386) or from that obtained for the Eastern cluster (GD = 55.25; P = 0.865). These results suggest that they probably are immigrants from the same external source population.

In order to make inferences about the direction of migration, we divided the sampling area into two halves that are separated by a strip of vegetation (see Figure 1). The two halves differ in the structure of the habitat; the Western side (comprising site W) is more homogeneous than the Eastern side (comprising sites C and E; see above). We can therefore estimate immigration rates (see the STRUCTURE results above) into the Eastern and Western sides of the sampled area excluding the external migrants (defined previously by the GeneClass exclusion test) in order to analyze the possible influence of landscape configuration on the dispersal pattern. The proportion of migrants into the Eastern side (14.38%) was significantly different from that estimated for the Western side (2.05%, G test, P < 0.001; Figure 1) indicating that there is an asymmetry in the migration, which appears to be predominantly in the West to East direction. Concomitantly, the proportion of individuals with ambiguous assignment is significantly larger in the Eastern side (26.92%) than in the Western side (7.4%, G test, P < 0.006; Figure 1), suggesting that admixture is stronger in the Eastern side.

The results obtained with TESS were very similar to those obtained with STRUCTURE; two genetic clusters were identified independently of the value used for the spatial interaction parameter ψ (between 0.1 and 0.9). Although we observed an influence on the proportion of each individual genotype that is assigned to each inferred cluster, the inferred number of clusters itself and the cluster to which each individual was assigned remained unaffected. We also carried out an analysis for studying genetic structuring at a local level within the Eastern and Western sampling sites using TESS. These analyses indicated that there is a lack of spatial substructuring within both sampling sites.

We also carried out separate Mantel tests for each sampling site. The test was significant for the Western sampling site (r = 0.18; P < 0.003), suggesting a restricted geographical dispersal at this geographical scale. In contrast, this test was non-significant for the Eastern sampling site (r = -0.056; P = 0.799).

### Inference of sex-specific dispersal patterns from multilocus genotypic data

We used two approaches to test for sex biased dispersal at regional scale. First, we looked at differences in the percentage of ancestry (*Q*) estimated by STRUCTURE between males and females [1]. No significant differences in *Q *were observed between sexes (t test; *P *= 0.27) at regional scale.

We also investigated sex-biased dispersal using the approach of Favre *et al. *[18] implemented in Fstat [19]. Neither the mean assignment index of individuals (mAIc) nor its variance (vAIc) were statistically different between sexes at the regional level (mAIc for females: 0.21, mAIc for males: -0.24, P = 0.16; vAIc for females: 6.12, vAIc for males: 6.23, *P *= 0.94). Also, both *F*_IS _and *F*_ST _were not significantly different between sexes at the regional scale (*F*_IS _for females: -0.0184, *F*_IS _for males: 0.0467, P = 0.11; *F*_ST _for females: 0.04, *F*_ST _for males: 0.018, *P *= 0.18). In sum, these four estimates are in agreement with the absence of a sex-biased dispersal pattern at the regional level; and are in concordance with the results of the STRUCTURE analysis.

**Figure 4:**
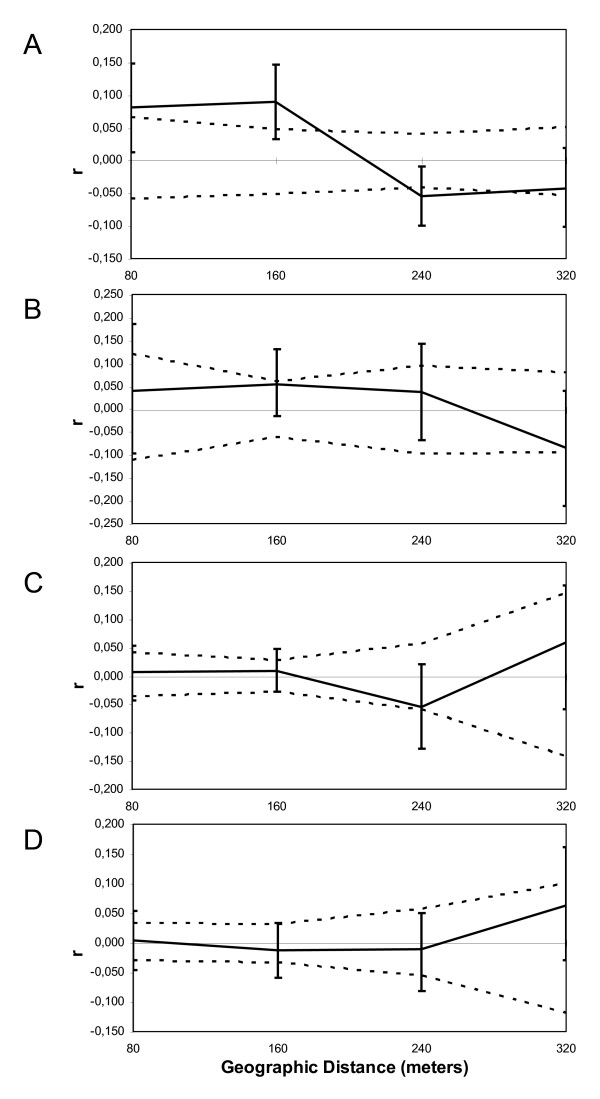
**Spatial autocorrelation analyses**. Spatial genetic structure autocorrelograms for females and males from the Western (A and B, respectively) and females and males from the Eastern sampling sites (C and D, respectively) showing the genetic correlation coefficient (r) as a function of geographical distance (only four distance classes of 80 meters each one are represented). The 95% confidence intervals for the autocorrelation coefficients (r) are also shown (broken line).

In order to test for sex biased dispersal at a small geographic scale (at the level of sampling sites), we calculated multilocus autocorrelation coefficients among males and among females within the sampling sites E and W (see Figure 4). Only females presented significant spatial autocorrelation at distances between 60 and 160 meters (P < 0.05) within the Western site (Figure 4A, B). On the other hand, neither the females nor the males from the Eastern sampling site showed significant spatial autocorrelation (< 320 m; Figure 4C, D), suggesting a lack of differences in the pattern of movements between sexes at this site. Overall, these results suggest a tendency for a slight philopatric pattern in females only at the local spatial scale and within the Western sampling site.

## Discussion

### Population structure and patterns of migration

The genetic structure and demography of local populations is closely linked to the scale and rate of dispersal [13]. In this study we make inferences about migration patterns at different spatial scales in the subterranean rodent *C. australis*. First, we detected significant genetic differentiation within the study area, indicating that the sampling sites do not form a panmictic unit. The Bayesian analyses showed that the three geographical samples were grouped into a Western (W) and an Eastern (E) clusters. Both Eastern and Western sampling sites are clearly two different subpopulations, while the Central sampling site represents a more or less even genetic mixture of genetic clusters E and W. Overall, the results indicate that dispersal within distances of less than 4 km is frequent. However, the rates of dispersal are not sufficient to avoid substructuring. These moderate dispersal capabilities suggest that small and seemingly minor disturbances may fragment habitat and subpopulations. Additionally, an asymmetric migration is detected among sampling sites, being predominantly in the West-East direction. More specifically, 14.38% of individuals from the Eastern sample site were originated in the Western genetic cluster. On the contrary, only 2.05% of individuals that were sampled in the Western site were identified as immigrants from the Eastern genetic cluster. In addition, the Eastern subpopulation presented a greater percentage of individuals whose origin is ambiguous. The assignment coefficients of these individuals suggest that they may be descendants from parents coming from different genetic clusters. On the other hand, the Western subpopulation is genetically much more structured as evidenced by its local *F*_*ST*_, which is higher than those of the Central and Eastern sampling sites.

Mantel tests provided no evidence for distance dependence of genetic structure at regional level, although it must be kept in mind that the statistical power of this test is relatively low here.

Migration at a small spatial scale (less than 1 km) seems to be enough to preclude spatial substructuring within sample sites. However, in the Western site there is an isolation by distance pattern and there is also evidence for sex biased dispersal, with males being more likely to disperse than females. The Eastern site, on the other hand, shows no signs of isolation by distance or sex biased dispersal. These results suggest that dispersal is more limited within the Western site, indicating a restriction in dispersal among individuals due to geographic distance.

The difference in dispersal patterns between Eastern and Western sampling sites seems to be explained by a combination of factors such as variation in landscape configuration (and the distribution of resources associated with this variation), density dependent factors (e.g. current individual densities), and differences in social structure and/or sex ratio.

In terms of landscape configuration we note that the sampling area is located near the Eastern limit of the species distribution [10]. Suitable habitat becomes scarcer in the West-East direction, showing a width between 450 and 550 m in the Western sampling site, and between 80 and 200 m in the Central and Eastern sampling sites (Figure 1). These differences in the extension of suitable habitat in both sites results from the current advance of grasslands over the coastal dune system, with the consequent fixation of the soil [11]. Besides the smaller extension of suitable habitat, the Eastern site is characterized by a patchier configuration than the Western one. The sand dune habitat is totally interrupted by grasslands at 1 km of distance from the Eastern site (principally due to human modifications), while this system has no interruptions for almost 10 km to the south-west of the Western site.

The population size in *C. australis *strongly depends on the availability of suitable habitat [10,11], which is more extended in the Western sampling site (Figure 1). The presence of small vacant habitat patches would facilitate the establishment of immigrant individuals in the East. Therefore, we expect that local turnover of individuals will be higher in the Eastern than in the Western side of the study area. These results are partially supported by the asymmetry in the migration and the proportion of migrants into the Eastern side. Additionally, previous studies in this species considering the occurrence of underground burrows have revealed significant lower individual densities in the Eastern than the Western sampling site (Mora *et al*., unpublished data). Possibly, these differences, in conjunction with social factors (e.g. highly territorial behaviour and competition for resources) would limit the availability of vacant habitat, making the establishment of immigrants more difficult in the Western site.

As we said previously, the high local turnover of individuals in the Eastern site of the study area could be reflected, more probably, by the lack of a correlation between genetic and geographical distances at this scale. However, we must also consider that small and unstable populations that are recurrently experiencing extinctions and recolonizations are unlikely to have reached equilibrium [20].

In this context, there are several studies in rodents showing the genetic consequences of dispersal among patchy populations at very low spatial scales [6,13]. Particularly, Schweizer *et al. *[13] showed high genetic structure among different vole subpopulations associated to a surprisingly fine geographical scale (lesser than 2.8 km) on a fragmented farm landscape, suggesting that minor discontinuities would have a great importance in determining the dispersal patterns in this species. Additionally, a further study on this last species using mtDNA and microsatellites for other populations and considering several spatial scales has additionally shown the importance of landscape discontinuities over the patterns of historical and current gene flow [21].

Among tuco-tucos, *Ctenomys rionegrensis *is one of the most impacted species within the genus, and like *C. australis*, is strictly limited to sandy soils. Particularly, this species occur on a highly fragmented habitat in a strongly restricted geographic range in Uruguay. Populations of this species are separated by very few kilometers (from 5 to 40 km among them), conforming a classic metapopulation system along the coast of the Uruguay River. Interestingly, the microsatellite genotype data showed that *C. rionegrensis *is strongly structured geographically, with subpopulations constituting distinct genetic units and, like *C. australis*, showing an absence of an isolation-by-distance pattern [22]. At this scale of the analysis, these authors hypothesized that stochastic processes, like genetic drift, might be more important in promoting genetic differentiation in *C. rionegrensis *than linear distances among subpopulations. A reanalysis of these data [23] suggests that the interaction between geographic distance and elevation could also explain the lack of isolation by distance pattern.

A metapopulation study carried out by Mora [11] in *C. australis *along a coastal portion of its distributional range (40 km between the Quequén Salado River and Claromecó stream) showed that patterns of occupancy in this species were highly associated to the occurrence of the larger and more continuous suitable patches at shorter distances to the coastline. In contrast, the highest proportion of unoccupied suitable patches was observed in the most fragmented habitat. In sum, these results showed the great importance of the patch size and the landscape discontinuities over the patterns of habitat occupancy at medium and low spatial scales (between 1 and 10 km) in this species.

Otherwise, there are very few works describing direct dispersal patterns in species of *Ctenomys *in the field [7,8,11]. In general, the genus *Ctenomys *is known as a low vagility group of species, due to their morphology, energetic consumption associated with excavating new burrows and their exposition to predators aboveground. For an ecologically closer species - *C. flamarioni *(adult body size between 300 and 400 g) - Stolz [24] reported individual movements larger than 250 m all over the year and on a continuous sand dune habitat. Earlier radio-telemetry studies in adults of *C. australis *showed that males were able to move larger distances than 160 meters on a continuous habitat in a period of 10 days (Mora et al., unpublished data). However, on the basis of this last study, we cannot affirm that dispersal is male-biased or that the movement capacity in this rodent is larger than the distances reported in its closer species *C. flamarioni *using direct estimates of dispersal (capture-mark-recapture, [24]).

Overall, the results suggest that stochastic events like genetic drift, in conjunction with differences in habitat configuration among sampling sites and the availability of propitious habitats, could probably be more important in structuring the *C. australis *subpopulations at fine scale than the linear distance among them. Similar conclusions have been suggested for other mammalian taxa that occupy fragmented landscapes [25,26]. However, minor differences in social structure among sampling sites could not be rejected as a possible reason explaining the genetic patterns observed here.

### Patterns of dispersal between sexes

Life-history traits (e.g. mating systems, parental care), and ecological features (e.g., landscape discontinuities, habitat patchiness, interspecific interactions) could be viewed as a complex set of variables that interact to influence philopatry in rodents [27-31].

If dispersal is male-biased, we expected that the level of genetic structure over the study area will be lower among males than among females. However, neither the immigrant proportions nor additional statistical tests based on *F*_ST_, *F*_IS_, mAIc and vAIc between sexes provided evidence for a significant male-biased dispersal rates at the regional level.

In this sense, the absence of different patterns of migration between sexes at regional level could be an artefact of the moderate number of migrants detected, or maybe in part due to different behaviours according to the spatial scale of analysis [32,33]. As was suggest by Goudet *et al. *[34], additional factors such as the number of loci available for analysis and the level of polymorphism of the molecular markers are likely to affect the power of these tests [19,34]. In this study, levels of polymorphism of the microsatellite loci were lower than that observed in other studies in subterranean rodents of the same genera [12,22,29,30], making it difficult to detect sex biased dispersal at regional scale (~ 4 km). It should be noted, however, that independently of any inter-sexual difference, the overall results suggest that dispersal in *C. australis *is not restricted at a regional spatial scale.

On the other hand, at the local scale in the Western site, the multilocus spatial autocorrelation analyses showed that males seem more likely to disperse than females. This pattern of dispersal is in agreement with the most common pattern reported in subterranean rodents [2,12], and other fossorial and surface dwelling rodents [13,28]. Other studies in *Ctenomys *report patterns of philopatry at local level considering small spatial scales [6,12,29]. Cutrera *et al. *[12], Lacey [29] and Fernández *et al. *[30] report the existence of male-biased dispersal patterns and stronger kin structure among females than among males in *Ctenomys talarum*, *Ctenomys sociabilis *and *Ctenomys flamarioni*, respectively.

Two major hypotheses intend to explain sex biased dispersal in mammals and birds [20]. As was suggested by Greenwood [31], sex biased dispersal could be described by the resource competition hypothesis, which predicts that the sex remaining at its birth site will be the one that benefits most from home-ground familiarity [see also [20]]. Male-biased dispersal is commonly expected in polygynous species occurring in habitats where the resources are distributed in patches, usually guarded by only a few males. Thus, differences in habitat requirements could be viewed as a one possible reason to explain sexual differences in dispersal behaviour for *C. australis *at the local level. The home range sizes in this polygynous species are notoriously larger in males than those of the females (Mora *et al.*, unpublished data); therefore, we expected that males should move longer distances than females.

On the other hand, sex-biased dispersal could be explained by the inbreeding avoidance hypothesis, which is based on the principle that the sex that incurs the maximum cost from inbreeding will be more likely to disperse [32]. Further, the local mate competition hypothesis [33] suggests that individuals disperse so that they will not have to compete with their relatives for mates, allowing them to increase their inclusive fitness.

According to Solomon [27], male-biased dispersal could be expected in polygynous species, where males show strong local mate competition, which results in pressure for male dispersal. In this context, *C. australis *are most likely a polygynous rodents that do not associate frequently with the opposite sex except for mating; they live alone in burrow systems and are very aggressive toward conspecifics [8,11]. Even though the individuals from the Western site showed a sex-biased dispersal pattern, the local *F*_IS _was not significant and near to zero (= 0.001), supporting the idea of a random mating system (most likely in accordance to the inbreeding avoidance hypothesis).

Consequently, each of these situations might select for increased dispersal [20]. It should be noted, however, that in some species of *Ctenomys *(e.g. *C. talarum*; see [12]) these dispersal patterns could vary slightly depending on ecological and density-dependent factors (e.g. level of individual aggregation, distribution of resources, sex ratio) and with the spatial scales studied.

On the other hand, no previous study describes the patterns of movements for different age classes in *C. australis*. In this study we found both mature as well as subadults individuals among the migrants, and because mature migrants could have moved during any stage of their life, differences in migration related to age classes cannot be easily inferred. According to Zenuto & Busch [8], we observed a common occurrence of subadults among migrants. Direct observations suggest that short-range natal dispersal of juveniles is also frequent in other species of *Ctenomys *[12,24]. This conclusion is in accordance with the most accepted dispersal theory for subterranean rodents, which predicts that once adults have established their territories and burrows, they move very little over the course of their lives, except for minor boundary changes [3,7].

## Conclusion

Several species have discontinuous or patchy distributions even at a local scale [13], and individual dispersal connects spatially distinct subpopulations. In relation to this, this study suggests that the habitat discontinuities (such as grasslands and other typical low interdune habitats) may have a greater impact on population differentiation than geographic distance among subpopulations. At the regional scale of this analysis (approximately 4 km), the habitat structure is rather heterogeneous and provides numerous effective potential barriers to dispersal, establishing a number of isolated clusters of individuals [11]. In addition, both population size and landscape configuration could be suggested as possible causes of the asymmetry in gene flow between Eastern and Western sites. Furthermore, genetic drift within local populations might enhance the genetic structure among populations, but this feature requires exploration in further studies. It should be noted, however, there might probably be other explanations which cannot be excluded by this study (e.g. low success in reproduction after immigration).

Due to the progressive urbanization, forestry and the impact of human activities during the last decades the habitat of coastal sand dunes is rapidly being lost [10,11]. The fixation and subsequent transformation of the dune habitat restricts the occurrence of this species, driving these populations to a situation of isolation and fragmentation. At the moment, human activities not only are reducing the proportion of suitable habitat, but also are generating barriers to gene flow among different subpopulations over the coastal dune system [11]. Demographic effects, such as vulnerability to stochastic disturbances, can be very harmful in isolated populations, and thus result in local extinctions [2]. Therefore, the current rate of landscape fragmentation could lead to an important reduction in the viability of populations of *C. australis *within this sand dune habitat. Future analysis over different landscape configurations and at different spatial scales will allow us to increase our understanding of the factors that model the complex processes of population differentiation and microevolution in subterranean rodents.

## Methods

### Study area and sampling design

Sampling was conducted in a sand dune habitat along the south-eastern Atlantic coast, localized at 15 km to the south-west of Necochea city (38°37'S; 58°50'W), Province of Buenos Aires, Argentina (Figure 1). Sampling was carried out during a two-year period, from April 2003 to April 2005 (avoiding the reproductive season between November and January, [8]). In this area, the sand dune habitat reaches altitudes ranging from 30 to 50 m above sea level, and depending on the geographical localization, the width of the coastal dunes can vary between 200 m and 15 km [8]. The vegetation over dunes is scarce; between 5 and 25% of the typical *C. australis *habitat is covered by natural grassland [8,9].

A total of 112 individuals of *C. australis *were captured from three sampling sites: a Central site (C) of 4 ha, an Eastern site (E) of 7 ha, and a Western site (W) of 8 ha. Sample sizes at each location are presented in Table 1. The two closest sites (E and C) are separated by 500 meters, whereas the two most distant ones (E and W) are separated by 3.8 km. The choice of these sampling sites was based on differences in habitat configuration. Thus, even though the richness of endemic plant species were similar over the capture positions at the 3 sampling sites (containing the same native dune species such as *Panicum racemosum*, *Hydrocotyle bonariensis *and *Achyrocline satureioidis*, [9,11]), these geographical sites showed markedly differences in habitat characteristics.

At this geographical scale (regional scale) the habitat is patchy, being interrupted by low inter-dune grasslands that constitute potential barriers to dispersal. The Eastern and Central sampling sites have a smaller extension of suitable habitat and are characterized by a more patchy configuration than the Western sampling site (Figure 1). The suitable habitat for *C. australis *in these two sites is surrounded by harder, deeper and more developed soils (not appropriate for the occupation of this species, [11]). In contrast, the Western sampling site is more homogeneous, and has no interruptions for almost 10 km to the south-west (see "*habitat fragmentation*" in results). Previous studies showed strong differences in the Normalized Difference Vegetation Index (NDVI, t = -26.08, p < 0.001) and the habitat heterogeneity (inferred as the differences in the NDVI variances, Levene's test, F = 7.43, p < 0.001) between the Eastern/Central area, regarding to the area including the Western sampling site (Mora *et al. *unpublished data). In spite of these differences, we carried out an independent analysis of habitat fragmentation. In order to quantify the differences in habitat configuration among sampling sites we employed an additional study of habitat fragmentation using a high resolution satellite images (Landsat ETM+) and a package of analysis available in the IDRISI software [35]. Firstly, we used a combination of remote sensing analysis and data on geographical position of individuals to determine the suitable habitat of occupancy for this species in each of the sampling sites (see [11]). The habitat classification allowed us to determine accurately the sand dune habitat availability for this species within each of the sampling sites. Then, we applied the fragmentation index (F = (n-1)/(c-1)) over each suitable habitat matrixes, as described by Monmonier [36]; where n is the number of different classes present in the kernel (or a measure of the environmental heterogeneity), and c is number of cells considered (or the kernel size). We used a kernel size of 5 × 5 pixels (150 × 150 meters). The degree of habitat fragmentation among sampling sites were compared using a Mann-Whitney test.

We obtained tissue samples (toe snips for subsequent DNA extraction and genetic analyses) from the captured individuals, who were live trapped with Oneida Victor N°0 snap traps (Oneida Victor, Inc., Ltd., Eastlake, OH, USA), with a rubber cover to avoid injuring animals (experience indicates that this procedure neither affects survival nor digging performance of individuals). After collection of tissue samples for genetic analyses, animals were immediately released back within the same burrow system where they had been originally captured.

Position of captures was recorded with GPS to determine their location. To determine different age classes among individuals we obtained detailed information about the reproductive status and body size of collected individuals. An open or plugged vagina and signs of pregnancy or postpartum pregnancy was indicative of sexual maturity in females (following [8]). The age class of males was inferred from an individual size (weight) distribution curve obtained by us from all the individuals that were captured in the sampling area during a period of three years. Details about the number of individuals of each age class and sex are shown in Table 1. All parts of the study involving live animals were approved by the National Scientific Council of Argentina (CONICET PIP 5838 2005/2006) and followed guidelines of the American Society of Mammalogists [37].

### DNA extraction

Tissue samples for DNA extraction were preserved in 95% ethanol and transferred to freezers at -70°C. Tissues were prepared and deposited in the collection of Laboratorio de Evolución, Facultad de Ciencias, Montevideo, Uruguay. Genomic DNA was isolated following a protocol modified from Miller *et al. *[38], involving treatment with sodium-dodecyl-sulphate (SDS) and digestion with proteinase-K, NaCl precipitation of proteins, and subsequent isopropylic alcohol precipitation of DNA.

### Polymerase chain reaction amplification and screening of Microsatellites

Primers of nine dinucleotide microsatellite loci developed from the Argentinean species *Ctenomys haigi *(Hai3, Hai4, Hai9, Hai11; [39]) and *Ctenomys sociabilis *(Soc1, Soc2, Soc5, Soc6, Soc8; [29]) that proved to be polymorphic in *C. australis *were surveyed. PCR amplifications were performed using fluorescently labelled primers and carried out in a reaction volume of 15 μl containing 1 unit of Taq Platinum Polymerase (Invitrogen), 3 μl of a total DNA solution 1/100, 2 mM MgCl2, 0.2 μM each primer, and 0.8 μM dNTPs (0.2 μM of each), using a "Rapidcycler" (Idaho Technology, Salt Lake City, Utah, USA). The thermocycling profile included an initial denaturing at 94°C for 10 min (polymerase activation), followed by 30-35 cycles of denaturing at 94°C for 30 s, annealing at 48-56°C for 30 s, extension at 72°C for 45 s, and a final extension at 72°C for 5 min. Relative concentrations of primers were adjusted by trial and error to create multiplexes. Final PCR fluorescently labelled product were analyzed with a capillary sequencer ABI3100 (MACROGEN, Inc., Korea). Considering the relative sizes of fragments, and the number of dye labels compatible with the capillary electrophoresis system we used the following four loci combination of multiplexing: Soc7/Soc8/Hai9; Soc2/Soc6; Hai3/Soc1; and Soc5/Hai4. Fragments were scored with the programs GENESCAN 3.0, GENOTYPER 2.5 (Applied-Biosystems).

### Statistical analyses

Genetic diversity within each sampling site was measured as the number of alleles per locus (At) and per sampling site (A), the percentage of loci that were polymorphic (%P), observed (Ho) and expected (He) heterozygosity [40], using ARLEQUIN 3.0 [41].

Analysis of linkage disequilibrium between pairs of loci and deviations from Hardy-Weinberg equilibrium were tested using ARLEQUIN 3.0 [41]. We used 1000 dememorization steps and 100000 iterations for the Markov chain implemented by the method of Guo & Thompson [42]. Sequential Bonferroni corrections for tests of linkage disequilibrium were applied to compensate for an inflating type-one-error [43].

We used the software Cervus 3.0 [44] to evaluate the presence of null alleles within each sampling sites, taking into account a cut-off point of 0.05. Pair-wise *F*_ST _between sampling sites were computed using Weir & Cockerham [45] estimator. Estimates of global levels of gene flow were calculated by the private allele method [46]. These analyses were also implemented using ARLEQUIN 3.0 [41]. Additionally, we used the hierarchical Bayesian method of Foll & Gaggiotti [47], as implemented by GESTE, to estimate population specific *F*_ST_s. This method estimates individual *F*_ST _values for each local population using the approach first proposed by Balding & Nichols [48]. Greater local *F*_ST_s values mean greater genetic structure of this particular population compared with the other ones. We used 10 pilot runs of 2000 iterations to obtain the parameters of the proposal distributions used by the MCMC. We also used an additional burn in of 5 × 10^5 ^iterations and a thinning interval of 50. All posterior probability estimates were obtained using a sample size of 20000.

In order to determine if there was a pattern of isolation by distance we carried out a correlation analyses between pair-wise genetic distances and linear geographical distances considering each pair of individuals [49,50]. We did this for both the regional (4 km) and local (within sampling sites) levels. A Mantel [51] test was used to assess the significance of the correlation between log-transform genetic and geographical distances. We generated 1000 permutations to create the null distribution of the *r *statistic, using the software POPTOOLS 2.6.2 [52].

We used the method of Pritchard et al. [16] implemented in the software STRUCTURE 2.1 to estimate the number of populations and assign individuals to them. We let K (number of clusters) vary between 1 and 6 and used the admixture model (which allows for the possibility that individuals may have mixed ancestry in more than one of the K populations) with correlated allele frequencies, as suggested by the software developers for closely related populations [53]. We conducted 6 independent runs for each K value. Preliminary runs showed that convergence was achieved after 3 × 10^6 ^iterations. We thus used this as burn-in and based the estimations on 3 × 10^6 ^additional iterations. We used Q > 0.7 as threshold for assigning individuals to populations. Individuals that were below this level were subjected to the exclusion test implemented in GeneClass [54] to determine if they could have originated from unsampled populations. For this propose we used the Bayesian method reported by Rannala & Mountain [55] and the simulation algorithm described by Paetkau *et al. *[56], both of them implemented in GeneClass. As suggested by Paetkau *et al. *[56], we used an exclusion threshold of 0.01.

We used GENALEX 6 [57] to calculate genetic distances (based on a stepwise mutation model and considering the allele sizes, see [58]) between excluded individuals and the approach of Piggott *et al. *[59] to determine if immigrants from unsampled populations originated from more than one population. This approach compares the average genetic distance [60] among the individuals from unsampled populations (individuals that were previously excluded with GeneClass) to a distribution of average genetic distances among randomly sampled nonimmigrants from each of the genetic clusters. If the genetic distance among the putative immigrants from unsampled populations was significantly greater than that among the randomly sampled nonimmigrants from each of genetic clusters defined by STRUCTURE, we considered that the immigrants were likely to have originated from more than one source population. We used a 1000 bootstrap replicates to calculate the distribution of average genetic distances within the populations identified by STRUCTURE, using the software POPTOOLS 2.6.2 [52]. Rough estimates of immigration rates were obtained by dividing the number of individuals identified as migrants by the total number of individuals sampled from each subpopulation as suggested by Rannala & Mountain [55], Paetkau *et al. *[56] and Manel *et al. *[5]. Individuals that could not be assigned to a population or excluded from all the sampled populations were not considered.

In order to detect regional and local substructuring within the sampling sites we also used the software TESS [61] that implements the method of François *et al. *[17]. We considered values of Kmax between 1 and 8 and ran 5 independent analyses for each one of them in order to verify convergence. We used a burn-in of 10^6 ^followed by 10^6 ^additional iterations. The interaction parameter (ψ) of the Hidden Markov Random Field was set to 0.6. The spatial Bayesian clustering algorithm implemented by TESS is supposed to perform better than STRUCTURE when genetic differentiation is low and when the genetic units are closely distributed in a continuous or quasi-continuous habitat [61].

Genetic inferences of sex-biased dispersal patterns were done using four statistical indices calculated by Fstat 2.9.3.2 [19]. This analysis was done excluding the smaller subadults (see Table 1). The first two indices are the traditional global descriptors of population structure, *F*_IS _and *F*_ST _[45]; the two others were based on an approach relying on individual genotypes (assignment index, AI), the mean of the corrected assignment index (mAIc) and the variance of AIc (vAIc; [18]). Assignment indexes estimate the probability that an individual genotype may have originated in the population from which it was sampled. The distribution of AIc is centered on 0; a positive value indicates a genotype more likely than average to occur in its sample (probably a resident individual), while a negative value indicates a genotype less likely than average (likely a disperser). Because immigrants tend to have lower AIc values than residents, and individuals of the dispersing sex will include both residents and immigrants, under sex biased dispersal scenario is expected that the sex which disperses most will have a lower mean AIc and larger variance than the more philopatric sex. A randomization approach implemented in Fstat [19] was used to test significance of these indexes between females and males for the regional study area.

In order to determine if there were differences in dispersal between sexes at a fine scale (within the sampling sites), we carried out a spatial autocorrelation analysis (SAA) on the genetic multilocus data using GENALEX 6 [57]. Due to a low and unequal number of the smaller subadults among sampling sites we preferred excluding these individuals for the autocorrelation analyses (see Table 1). This method provides a measure of genetic similarity between pairs of individuals, whose geographic separation falls within some specified distance range, allowing an effective means of evaluating the genetic consequence of dispersal over these smaller spatial scales (see [60]). Different rates of dispersal by males and females are expected to result in stronger spatial autocorrelation among individuals of the more philopatric sex [59,62]. We carried out these analyses on the Western and Eastern sampling sites, and tested the significance of each SAA using a nonparametric permutational procedure described in detail by Peakall & Smouse [57]. Basically, the significance tests were computed for each distance class by comparing the observed value of the coefficient *r *with those obtained from 999 spatial permutations of individuals belonging to the site of interest. Then, the observed data were added as the 1000^th ^permutation, on the null hypothesis that our data were genetically random with respect to geographic position. The 95% confidence intervals over the correlograms were obtained as described by Peakall & Smouse [57]. This analysis was not carried out for the Central sampling site due to its very small sample size.

## Authors' contributions

MSM designed and conceived this study. MSM and FJM collected the genetic data, and with OEG, MJK and EPL performed all the genetic analysis and drafted the manuscript. All authors read and approved the final manuscript.
